# Sex-specific gene expression in the mosquito *Culex pipiens* f. molestus in response to artificial light at night

**DOI:** 10.1186/s12864-015-2336-0

**Published:** 2016-01-05

**Authors:** Ann-Christin Honnen, Paul R. Johnston, Michael T. Monaghan

**Affiliations:** Leibniz-Institute of Freshwater Ecology and Inland Fisheries (IGB), Müggelseedamm 301, 12587 Berlin, Germany; Freie Universität Berlin, Königin-Luise-Straße 1-3, 14195 Berlin, Germany; Berlin Center for Genomics in Biodiversity Research, Königin-Luise-Straße 6-8, 14195 Berlin, Germany

**Keywords:** RNAseq, Diptera, Culicidae, Gene expression, Transcriptome, ALAN, LED

## Abstract

**Background:**

Artificial light at night (ALAN) is a typical feature of urban areas and most organisms living in urban or suburban habitats are exposed to low levels of ALAN. Light is one of the most important environmental cues that organisms use to regulate their activities. Studies have begun to quantify the influence of ALAN on the behavior and ecology of organisms, but research on the effects at the molecular level remains limited. Mosquitoes in the *Culex pipiens* complex (Diptera, Culicidae) are widespread and abundant in urban areas where they are potential disease vectors. It is thus of particular interest to understand how ALAN may influence biologically and ecologically relevant traits.

**Results:**

We used RNAseq to evaluate the transcriptome response in a *Cx. pipiens* f. molestus laboratory population that was exposed to near-natural light conditions (light:dark L16:D8 hours, “control”) and ALAN conditions with 3 h of constant low-level light at night (L16 + L_low_3:D5 hours, “low-light”). The resulting transcripts were mapped to the reference genome of the closely related *Culex quinquefasciatus*. Female expression patterns differed significantly between control and treatment conditions at five genes although none showed an absolute fold change greater than two (FC > 2). In contrast, male expression differed at 230 genes (74 with FC > 2). Of these, 216 genes (72 with FC > 2) showed reduced expression in the low-light treatment, most of which were related to gametogenesis, lipid metabolism, and immunity. Of the 14 genes (two with FC > 2) with increased expression, only five had any functional annotation. There was a pronounced sex-bias in gene expression regardless of treatment, with 11,660 genes (51 % of annotated genes; 8694 with FC > 2; 48 % of annotated genes) differentially expressed between males and females, including 14 genes of the circadian clock.

**Conclusion:**

Our data suggest a stronger response to artificial light by males of *Cx. pipiens* f. molestus than by females, and that a wide range of physiological pathways may be affected by ALAN at the molecular level. The fact that differences in gene expression appear to be sex-specific may have a strong influence at the population level.

**Electronic supplementary material:**

The online version of this article (doi:10.1186/s12864-015-2336-0) contains supplementary material, which is available to authorized users.

## Background

Artificial light at night (ALAN) is a typical characteristic of the habitat for organisms living in urban and suburban environments. Along with temperature, light is one of the most important environmental cues for all living things [1], playing a key role in regulating daily (i.e., foraging, photosynthesis) and seasonal (i.e., migration, diapause) activity. As a result, changes to light regimes can play a major role in the ecology and physiology of a wide range of organisms and ecosystems [1, 2]. The addition of ALAN, whereby light of a different spectrum and intensity to natural light is present during naturally dark phases, has been demonstrated to affect metabolism [2, 3], behaviour [4] and circadian clock regulation [5–8] in numerous organisms. Nonetheless, our understanding of the effects of artificial illumination remains limited to a relatively small number of species and ecosystems.

Mosquitoes (Diptera, Culicidae) are an ecologically important group of insects and several species occur in urban and suburban areas in close proximity to humans, where they are exposed to artificial light at night [9–11]. The *Culex pipiens* complex is a widespread and abundant group of mosquitoes [12] and blood-feeding females act as vectors of vertebrate diseases (e.g. West Nile virus, avian malaria) [13]. Male *Cx. pipiens* respond directly to light cues and the onset of low light by becoming active and starting to swarm [14]. Although the potential impact of light on circadian rhythms has been studied [8, 15, 16], there remain gaps in understanding whether ALAN evokes changes at the molecular level on processes that may not be directly involved with the circadian clock. Most *Cx. pipiens* research is focused on females because of their role in disease transmission; however, studies of population-level responses to environmental change must also consider the consequences of artificial light for males. There are pronounced differences in the behaviour and physiology of male and female mosquitoes, e.g. food preference and daily activity [8]. At the molecular level, the only study of which we are aware that reported sex-biased gene expression patterns in adult mosquitoes analysed the malaria vector *Anopheles gambiae* [17].

A number of physiological pathways could potentially be affected by environmental cues from artificial illumination at night. The genes of the circadian clock are expected to be affected by light because the blue-light receptor cryptochrome-1 [18, 19] receives environmental light cues that are used in clock synchronisation [13]. Rund et al. [8] suggested that a number of additional genes are expressed in diel patterns in response to the surrounding light environment. These genes relate to metabolic detoxification, immunity, and nutrient sensing (e.g. glutathione-S-transferase, serine protease inhibitor and *takeout genes*, respectively) [8]. This implies that physiological processes not under direct circadian control may nonetheless be influenced by artificial light, with effects discernible at the molecular level.

**Fig. 1:**
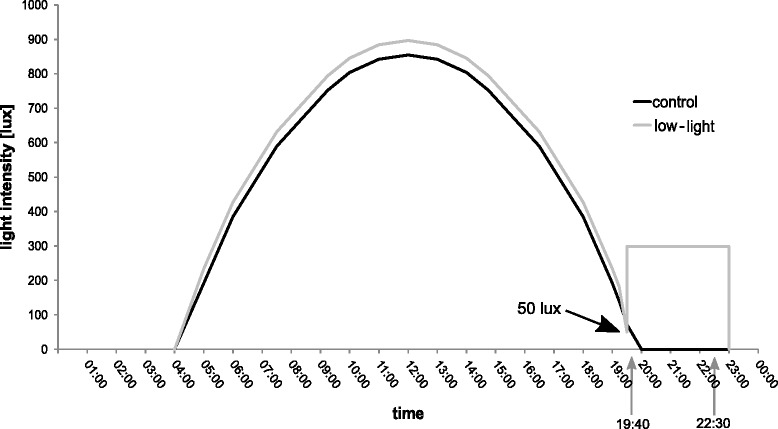
Light regime throughout the experiment. Light intensities, in lux, against clock time throughout the day. The dark line depicts “control” (L16:D8h) and the grey line refers to “low-light” regime. *Arrows* delimit sampling times that were used as biological replicates after preliminary alanysis (see text). Before “low-light” (L16 + L_low_3:D8h) regime reached the constant additional light at night level (300 lux), light intensity smoothly droped to 50 lux. “Control” light regime decreased to darkness uninterrupted

Here we tested how exposure to low levels of artificial light at night alters gene expression in *Cx. pipiens* f. molestus by combining controlled laboratory experiments with a near-natural light regime (“control” consisting of Light(L)16 h:Dark(D)8 h) compared to an ALAN light regime (“low-light”, L16h:L_Low_3h:D5h; Fig. 1) and transcriptome (i.e., the complete set of transcripts in a cell, and their quantity [20]) analysis using RNAseq. The genome of the closely related *Cx. quinquefasciatus* was used as a reference in our analysis.

*Culex pipiens* includes two forms (ecotypes) with partially sympatric distributions, pipiens and molestus. Females of the pipiens form depend on blood meals (anautogeny), and males build mating swarms, whereas females of the molestus form are autogenous and mating can take place in confined spaces. These characteristics make the molestus form highly suited to rearing in the laboratory, allowing for testing of different light regimes under otherwise constant conditions.

Our analysis of the transcriptome revealed a strong sex-bias in gene expression regardless of treatment (approximately 50 % of annotated genes). Females generally exhibited little response to ALAN treatment (up to 5 differentially expressed genes), whereas males exhibited a pronounced response (up to 230 genes), with most changes being reduced expression across a range of gene functions. Our findings suggest that a wide array of physiological pathways may be affected at the molecular level by ALAN.

## Results

Sequencing of pooled samples (seven individuals per pool, whole bodies) yielded a total of 195.5 million 100-bp paired-end reads (~400 million total reads) giving an average of ~24.5 million read pairs per sample across eight samples (range 21,589,314 – 28,382,099 read pairs). The reads were mapped to the *Culex quinquefasciatus* reference genome assembly version CpipJ1.21. Approximately 37–57 % reads aligned to the reference (37–45 % in males, 55–57 % in females) with 17,573 of 22,985 genes receiving at least 1 aligned read. Further annotations, including gene ontology (GO) terms, were obtained from the UniProt-GOA *C. quinquefasciatus* proteome annotation (see Methods). Because gene expression may show temporal variation [8], we first included treatment and sampling timepoint as factors using negative binomial generalized linear models implemented in the R package DESeq2 version 1.8.2 [21]. Genes were considered differentially expressed when the *p-*value was <0.05. All *p-*values reported in the text refer to the *p-*values adjusted after Benjamini-Hochberg as implemented in DESeq2. Of theses we additionally report the number of genes that showed an absolute 2-fold change in expression (FC > 2).

Only two genes were differentially expressed (FC > 2) according to both treatment and sampling timepoint (in males). Based on this, we ran a second model that treated the two timepoints as replicates and included only treatment as a factor. The results of this second model (i.e. with two replicates per sample) are reported below.

### Differential expression testing — artificial light treatment

**Fig. 2:**
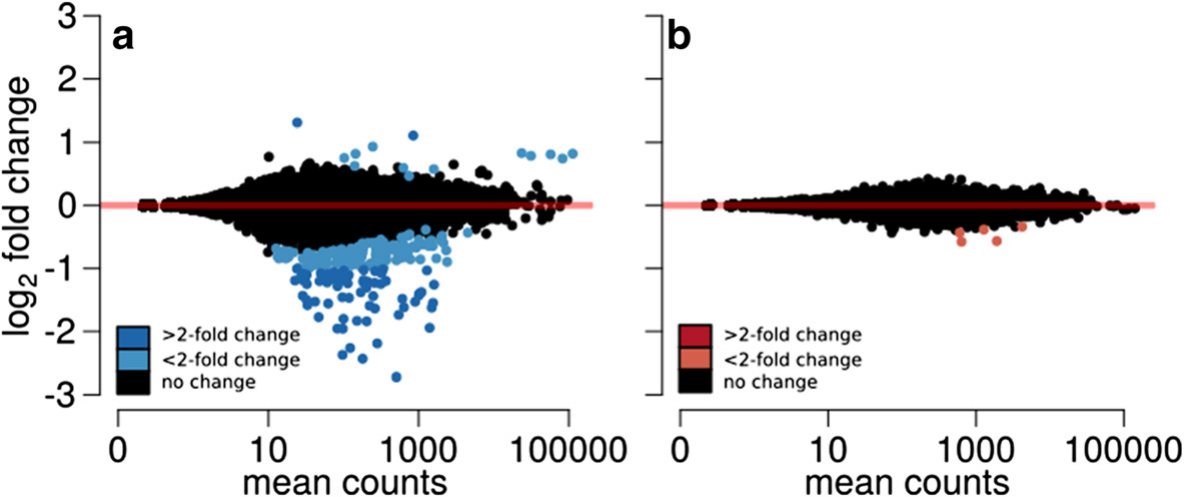
Ratio average plots of differentially expressed genes between treatments. Differentially expressed genes between treatments separately for (**a**) males and (**b**) females. The different colours refer to the differences in fold changes (FC). Positive and negative fold changes indicate genes with treatment- and control -biased expression respectively

In response to light treatment 230 (74 > 2FC) genes were differentially expressed in males. Of the genes with >2FC, two were more highly expressed in the “low-light” condition, while 72 genes were more highly expressed in the “control” (Fig. 2a; Additional file 1).

In contrast, the change in gene expression of females never exceeded the >2FC cut-off although five genes had a *p*-value < 0.05 (Fig. 2b; Additional file 2).

In males, a striking signature of gametogenesis-related gene expression was identified in the response to artificial light, where expression of genes involved in DNA replication, mitosis, meiosis, spermatogenesis and germ cell proliferation was reduced in artificially lit conditions (Additional file 1). This included a substantial portion of the DNA replication machinery, i.e. all six DNA replication licensing factors (*mcm2-7*); the components of the origin recognition complex *geminin*, *orc1* and *cdt1*; DNA polymerase; and an ortholog of *fizzy* which regulates transition to meiosis. There was clear coordinate expression of several genes with crucial roles in spermatogenesis, such as *ance* [22] and *importin alpha* [23]. This includes the major regulator of zygotic genome activation *smaug*, and the cognate activating kinase *pan gu* (Additional file 1). Ten additional genes were downregulated that are orthologs of genes that exhibit germ-line stem cell bias in *Drosophila* [24], as well as a gene encoding a putative uncharacterized protein that possesses a meiosis arrest protein family domain (IPR024768, Additional file 1). Gene ontology (GO) term enrichment analysis also highlighted the signature of gametogenesis with enrichment of GO terms related to gametogenesis (Additional file 3) including “cellular process involved in reproduction in multicellular organism” (GO:0022412), “gamete generation” (GO:0007292), “germ cell development” (GO:0007281), and “regulation of cell cycle” (GO:0051726).

Differential expression of immune genes was also observed, with reduced expression in the artificial light treatment of genes encoding transferrin and orthologs of the *Drosophila* genes *yellow-f* and *yellow-h* which encode dopachrome conversion enzymes that participate in the melanisation response [25]. Three genes encoding proteins with functions in lipid metabolism and transport were also differentially expressed in males in response to artificial light: apolipoprotein and two genes encoding sterol acyltransferases (Additional file 1).

### Differential expression testing — sex-biased gene expression

**Fig. 3:**
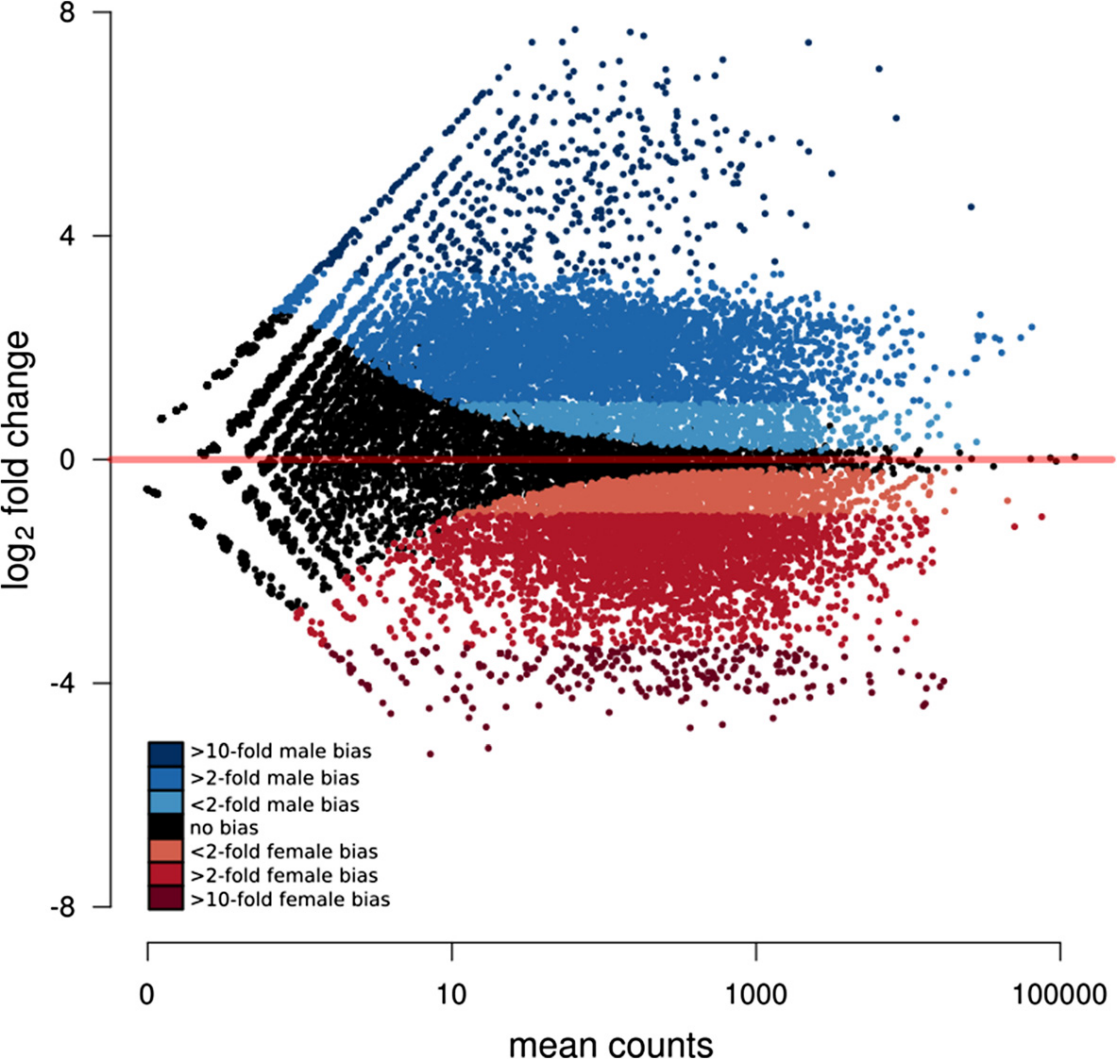
Ratio average plot of differential expression between males and females. The different colours refer to the differences in fold change (FC) sex bias; blue for males, red for females. MB, male-biased; FB, female-biased

Simultaneous analysis of males and females was performed to test for the main effect of sex across all samples. Independent of treatment, 11,660 genes (ca. 51 % of annotated genes) were differentially expressed between males and females (Fig. 3; Additional file 4).

Of these, 8694 genes exhibited at least a 2-fold sex bias (48 % of annotated genes). Fourteen genes related to circadian clock function were differentially expressed between males and females (Table 1). The genes *photolyase, disc overgrown protein kinase* and *timeout/timeless-2* were male-biased while the rest were female-biased (Table 1).

**Table 1:** circadian clock and related genes differentially expressed in females (negative values) and in males (positive values)

	Gene name	Gene id	Fold change	SE
Canonical clock genes	Clock^a^	CPIJ002146	1.811	0.177
	Clock^a^ - continued	CPIJ002147	1.879	0.130
	cryptochrome-1	CPIJ009455	1.310	0.126
	photolyase^b^	CPIJ017734	-1.969	0.191
	Duplication of cryptochrome-2^c^	CPIJ015481	1.749	0.152
	cryptochrome-2	CPIJ018859	1.528	0.111
	cycle^d^	CPIJ014938	1.512	0.195
	period	CPIJ007193	1.561	0.166
	timeless protein	CPIJ007082	1.666	0.117
Genes related to circadian rhythm	timeout/timeless-2^e^	CPIJ000660	−2.586	0.193
	par domain protein^f^	CPIJ014920	1.729	0.135
	discs overgrown protein kinase^g^	CPIJ003503	−1.481	0.090
	hypothetical protein	CPIJ016941	1.279	0.089
	mck1	CPIJ006114	0.384	0.121

^a^circadian locomotor output cycles kaput protein (contains artificial break point)

^b^annotated in reference as cryptochrome 1

^c^annotated in reference as cryptochrome 1

^d^circadian protein clock/arnt/bmal/pas

^e^paralog of timeless

^f^homolog of par domain protein 1 (pdp1) in *Drosophila melanogaster*

^g^alternative name: doubletime

We compared our overall results to those of Baker et al. [17] who presented a gene expression atlas addressing sex- and tissue-specificity for the mosquito *An. gambiae*. They reported 1487 female-biased and 1226 male-biased genes, belonging to 1351 and 1030 ortholog groups, respectively. By comparing the expression of genes belonging to ortholog groups that were conserved in *An. gambiae*, we found that 61 % (female) and 65 % (male) of these groups showed sex bias in *Cx. pipiens* (based on *p*-value < 0.05 and >2FC). Analysis of gene ontology terms that were specifically enriched in each sex showed an overlap of a single GO term with *An. Gambiae*, “ion transport” (GO:0006811) in males (Additional file 5).

## Discussion

Despite increasing recognition of the role of ALAN in ecology and behavior [1–3], our understanding of how it affects organisms at the molecular level remains limited. Here we assessed the effects of artificial light on *Cx. pipiens*, a widespread and abundant mosquito that is prominent in many urban habitats, by examining changes in gene expression in the entire body. This is the first study to provide data on sex-specific gene expression in adult *Culex pipiens* f. molestus. The majority of studies on mosquitoes focus on females due to their role as disease vector.

We used *Cx. q*uinquefasciatus genome as a reference to map our data. The only other transcriptomic study of *Cx. pipiens* f. molestus to date found that the relationship between species was sufficiently close to obtain reliable mapping results [26]. Both species belong to the *Cx. pipiens* complex. They are known to be able to hybridise and the species status of *Cx. quinquefasciatus* remains debated [13, 27, 28]*.* This study, however, focussed on searching for ecotype-specific divergence between genes in samples of mixed sexes [20].

The experimental design of our study focused on separate analysis of sexes. There was a very strong sex bias in expression, and males in our laboratory population exhibited a much more pronounced response to the treatment mimicking artificial light at night (“low-light”), with many of the affected genes having functional annotation. This study may serve as foundation for future work by providing whole-body transcriptome data for a widespread mosquito and suggests that ALAN can affect a broad range of physiological pathways at the molecular level. Although we are aware that gene expression varies among tissues [8, 16, 17] we chose this approach because our goal was to obtain an overview of processes potentially affected by artificial light at night. This was done at the expense of tissue-specific responses, but we believe that our results provide an important starting point for more detailed studies concerning gene expression in separate tissues, different developmental stages or different light sources and regimes.

The finding that males and females are affected to a different extent fits with the only other published study of sex-specific gene expression in mosquitoes [17] and suggests possible implications for reproduction biology and, consequently, population-level impacts.

Our experiment was designed to mimic artificial light of the kind generated by street and other outdoor lighting, where the normal transition from natural light (during the day) to darkness at night is altered by an abrupt switching on of light for a period of constant brightness. Veronesi et al. [14] found that certain light intensity thresholds (e.g. 5 lux for *Cx. pipiens*) function as cues for commencing or ceasing activity. Although the light regime in the treatment never fell below this threshold, our light regime still provides a cue for anticipation of the onset of darkness by its design to mimic the natural rise and fall of light intensity. In contrast, no cue was provided that allows anticipation of the abrupt switch to “artificial low-light” (300 lux in our experiment, an increase of a factor of ~6). We therefore believe the experiments measured a response to artificial light rather than comparing a short day to a long day. However, our choice to deprive individuals of light cues for 48 h prior to the experiment, and thus prohibiting synchronisation to the ambient light environment, constitutes an acute change relative to the darkness experienced before. As a nocturnal species, *Cx. pipiens* is naturally exposed to varying, albeit low, light intensities produced by moonlight. Studies have suggested that lunar cycles can influence the activity and biting propensity of mosquitoes [29, 30]. There were no cues regarding the lunar cycle in our laboratory setup. Our results are thus likely to be the response to artificial light at night. However, the presence of moonlight in combination with artificial light may produce a different gene expression profile in natural populations, which remains to be addressed in future studies.

### Male response to artificial light at night

The response to artificial light at night in males was primarily detected as reduced expression levels of a number of genes in “low-light” treatment conditions, i.e. in males exposed to artificial light at night instead of the (laboratory-simulated) natural progression from daylight to darkness. These down-regulated genes were mainly related to gametogenesis, immunity and lipid metabolism. There is a scarcity of knowledge about the effects of artificial light on mosquitoes. Under natural conditions, decreasing light triggers activity in nocturnal mosquitoes. Individuals begin the search for food and mates, and males begin to swarm. We may speculate that the expression of genes involved in gametogenesis should increase as light levels decrease, but that artificial light inhibited this here. Our findings are preliminary, and we are not aware of any similar studies, so this remains a hypothesis to be tested in the future. Genes involved in lipid metabolism comprised another important group of genes that were less expressed under ALAN. The last food uptake was 12 h prior to sampling which could mean that the carbohydrate reserves had been used up. It may also mean that males in artificially lit environments were less active. Further work should address whether the observed changes in expression of genes related to lipid metabolism are caused by light-mediated reduced activity or a sign of usage due to starvation. Genes involved in immune response also exhibited lower expression. Some immune genes are known to be rhythmically expressed in *An. gambiae* [8, 16], and this might also be the case for *Cx. pipiens.* Despite the evidence for the negative influence of artificial light on immune genes and given their cyclic expression patterns, future studies with a 24-h sampling scheme could provide important insights that enable us to fully understand how these two processes interact.

It is well known that some genes exhibit cyclic expression over time [8]. Our initial analysis using ‘treatment’ and ‘timepoint’ resulted in only two differentially expressed genes over time and in response to treatment (in males). Neither of, these genes were detected as differentially expressed when the factor ‘timepoint’ was removed from the model, thus our results using only ‘treatment’ as a factor present a robust estimate for the gene expression level response to the low-light treatment, although this comes at the expense of detecting temporal differences that may occur in the response to light treatment. Future experiments using extended time series sampling under different light regimes may provide more insight into temporal changes in response to ALAN.

Of the genes more highly expressed in the low-light condition in males, the majority encoded conserved hypothetical proteins, i.e. have no clearly defined function. Only the one of the two genes with FC > 2 was annotated, namely as 4-coumarate-CoA ligase 1 (Additional file 1: Table S1). To date, the function of this gene is not known in mosquitoes.

### Female response to artificial light at night

It was striking that the different light regimes did not induce detectable changes in gene expression in female *Cx. pipiens*. One biological explanation is that females in both treatments were inseminated and this may have played a role. Male accessory gland secretion is a powerful modulator of female behaviour and activity [31], which might potentially render females insensitive to light at night. This could have implications for biting propensity, as accessory gland secretion can trigger ovulation and oviposition behaviour. An evaluation of the effect of light at night at different life stages (e.g., virgin, inseminated, or after oviposition) would provide important insights. However, females often mate soon after emergence [32] and thus it is reasonable to assume that the vast majority of adult females in nature are inseminated at a given point in time.

### Sex-biased expression

Half of all genes were differentially expressed in males and females, indicating a strong sex-specific pattern of expression regardless of the light treatment. Sex-biased differences in gene expression are known to occur in a number of species and sexual dimorphism (in morphology, behaviour and physiology) is believed to be a main driver of this [33]. Our findings were similar to those of a recent study of the mosquito *An. gambiae* that reported 72 % of genes to show sex-biased differential expression in whole bodies [17]. In *Drosophila*, approximately 50 % of genes are sex-biased [34]. By determining ortholog relationships between the sex-biased genes from our study of *Cx. pipiens* and those in *An. gambiae* [17] we found a larger overlap of sex-biased genes in males compared to females. This pattern could be explained by greater conservation of male-biased gene expression between *Anopheles* and *Culex*. Alternatively, this could reflect differences in ecology of females from these two species. *An. gambiae* is an obligate blood feeder whereas *Cx pipiens* f. molestus displays facultative autogeny (a blood meal is not essential). Furthermore, the present study sampled individuals reared on a sugar diet whereas *An. gambiae* data were derived from blood-engorged females and sugar-fed males [17], potentially contributing to differences in female-biased gene expression.

Among the genes that were differentially expressed in males and females were the circadian clock genes. Although clock-gene expression varies among tissues and our approach was a whole-body sampling, some notable findings suggest areas for more research on the effect of artificial light on clock genes. Male-biased genes related to the clock were generally much more highly expressed compared to females, in which genes were only slightly (although significantly) up-regulated. This suggests that the internal circadian clock system may either be influenced differently in males and females or is inherently different between the sexes, as is the case in *Anopheles gambiae* [35]. To our knowledge it has not been tested whether this sex-specific circadian rhythm is the case in *Culex pipiens*. The highly expressed male-biased genes included *photolyase*. This is a domain of the cryptochrome-1 protein which contains two light-harvesting cofactors and is mainly responsible for DNA repair after UV- and blue-light exposure [36]. *timeout/timeless-2* is a paralogue of circadian clock gene timeless and is involved in chromosome stability and light entrainment [37]. *disc overgrown protein kinase,* in *Drosophila* also referred to as *doubletime*. It phosphorylates the period protein and thus contributes to circadian rhythmicity [38]. All of these genes are involved in the perception of light and relate directly to circadian clock function, suggesting a greater potential for the low-light treatment to influence males compared to females, in agreement with our overall findings. Further underlining this potential sex-specific influence is the fact that we found the gene *cryptochrome-1* to be more highly expressed as a response to ALAN. The presence of light clearly changed the expression of the gene of paramount importance in synchronisation of the circadian clock to the environment and clock-controlled processes.

## Conclusion

In this study we addressed whether artificial light at night similar to that prevalent in urban and suburban settings would affect gene expression in the mosquito *Culex pipiens* f. molestus. Artificial light at night elicited responses at the molecular level with differing responses in males and females. In males, a significant reduction in expression occurred in genes related to gametogenesis, lipid metabolism and immune response. In contrast there was almost no differential gene expression in females. Taken together these changes potentially lead to dramatic shifts in population dynamics, as reproduction is directly influenced. This might lead to shifts in feeding behaviour of the females, as the search for a blood meal is associated to insemination, and hence vector capacity.

## Methods

Mosquitoes used in the experiment originated from a laboratory colony established in 2012 and were reared in a light(L):dark(D) regime of L16h:D8h (hereafter “control”) in a climate chamber with temperature maintained at 26 ± 1 °C and relative humidity at 60–90 %. Adults were kept in mesh cages (60 × 30 × 30 cm) and fed with ~ 10 % saccharose solution offered on cotton pads ad libitum. Light was provided by cool-white LEDs (LED flex SMD, 24VDC, 24 W, 1A, 60 LEDs/m, 500 cm, cool-white single chip, Barthelme GmbH & Co. KG, Nuremberg, Germany). Seven strips consisting of 48 LEDs each were attached to a board (88 × 34 cm) and suspended horizontally over the cages. Light levels in the colony were controlled with custom-made software based on the LabView v 8.5.1 runtime environment (National Instruments Germany GmbH, Munich, Germany). We specified voltage at 15 timepoints over a 24-h period (and at 19 timepoints in the low-light treatment, see below) to which the software fit a hermite spline curve. The result was a smooth change of voltage, and thereby light intensity, over each 24-h period. This allowed us to produce a near-natural light regime with gradually changing light intensities throughout the day (dark: 0 lux, mid-day: 855 and 897 in control and low-light regime, respectively; Fig. 1).

After pupation, individuals were placed into cages and adults were allowed to emerge and mix. We therefore assume that all females were mated. Adults were between 1–8 days old when they were removed from cages with an aspirator and placed in continual darkness for 48 h. This was done to uncouple mosquitoes from the light regime under which they were reared and thus prevent the onset of any processes arising from light-induced anticipation of the time of day. A 10 % saccharose-solution was available ad libitum for the first 36 h and feeding was stopped 12 h prior to the start of the experiment in order to avoid inflated expression of genes involved in digestion relative to other physiological processes. After the 48 h of darkness, adult individuals were exposed to three “days” in one of two different light regimes: “control” (L16h:D8h; the same conditions were used for rearing) and “low-light” (L16 + L_low_3h:D5h). The low-light treatment (low-light relative to the maximum light intensity) consisted of a 16-h day as in the rearing cage (described above) followed by a sudden increase to ~300 lux for three additional hours at sunset (Fig. 1). The experiment started on day 1 at 04:00 in both treatments and seven adults of each sex were sampled after 3 days at two timepoints (19:40 and 22:30) for each treatment. Samples were immediately placed in liquid nitrogen, resulting in eight samples. Each of the eight samples consisted of seven pooled individuals (either male or female). “Control” light intensity during sampling was 0-25 lux while “low-light” light intensity was ~300 lux.

Data were analysed first by considering these as independent timepoints and then by combining these and treating timepoints as replicates (see below).

Gene expression levels related to specific metabolic processes can differ among tissues [8, 17]. Because no data on tissue-specificity were available for *Cx. pipiens* f. molestus, we chose to examine whole bodies in order to investigate whole-organism effects of additional light at night across as broad a range of molecular processes as possible. A disadvantage of our approach was that any tissue-specific differences could not be detected; however, our aim was to gain an overview of gene expression and limit any risk of overestimating the biological significance of results based on tissue-specificity.

RNA was extracted by first adding TRIzol® Reagent (Ambion®, Invitrogen, Carlsbad, USA) to the samples. The tissue was then homogenised on ice with an ULTRA-TURRAX® disperser (IKA®, Staufen, Germany). All remaining steps were carried out according to the manufacturers’ protocol (Ambion®, Invitrogen, Carlsbad, USA) and the RNA-pellet was dissolved in 50 μl RNase-free water (Carl Roth GmbH und Co. KG, Karlsruhe, Germany). The construction of 8 TruSeq cDNA libraries and sequencing on the Illumina HiSeq2000 platform for 207 cycles was performed by LGC Genomics GmbH (Berlin, Germany). The resulting 100-bp paired-end reads are available from the NCBI SRA under BioSample accession PRJNA257052.

The untrimmed read pairs were mapped to the *Culex quinquefasciatus* reference genome version CpipJ1.21 using the unmasked DNA assembly (3171 supercontigs, totalling 580 Mb, with a Contig N50 of 28 Kb and supercontig N50 size of 486.76 Kb) and corresponding gtf annotation from ensembl metazoa in conjunction with RSEM v1.2.12 [39] and Bowtie 1.0.0 [40]. Bowtie was executed using the default parameters recommended for use with RSEM [39]. Further annotations, including gene ontology terms, were obtained from the UniProt-GOA *Cx. quinquefasciatus* proteome annotation (ftp://ftp.ebi.ac.uk/pub/databases/GO/goa/proteomes/). RSEM expects paired reads of uniform length and accounts for quality scores in its statistical model, although it usually does not significantly improve quantification accuracy over a reduced model when using reads with an illumina error profile [39]. Reads were therefore not quality-trimmed.

Differential gene expression in response to the low-light treatment was determined on raw read counts using DESeq2 v1.8.2 in R. We initially specified both treatment and sampling time in the model (design formula: “design = ~treatment + timepoint) and then ran a second analysis using only treatment (“design = ~treatment”). Both models were tested for males and females separately. Sex-specific gene expression has been observed in a range of organisms [17, 33, 41]. We therefore tested for sex-specific differences across all samples using DESeq2 as described above. We specified the design formula as “design = ~ sex + treatment”. This allowed for a contrast of levels for each factor.

DESeq2 uses the negative binomial distribution and a shrinkage estimator to determine variance–mean dependence in mapped read counts and a conditional test for differential expression [26]. Genes with an Benjamini-Hochberg adjusted *p*-value < 0.05 (implemented in DESeq2) were considered to be differentially expressed. We also report numbers of genes with an absolute fold change greater than 2 (FC > 2) for reference to other studies. Over-representation of Gene Ontology (GO) terms in groups of differentially expressed genes was determined using the GOstats v. 2.14 package [42] for R, by means of a hypergeometric test with a *p*-value of 0.05 and the GO terms from the CpipJ1.21 proteome annotation as the gene universe. Gene orthology data were obtained from OrthoDB v. 8 [43] and sex-biased *Anopheles gambiae* gene expression data [17] were downloaded from the Sebida database [44].

## Data accessibility

The datasets supporting this article are freely available on the website of the research group: http://monaghanlab.org/data/cxpip_rnaseq/.

## Additional files

Additional file 1: Table S1. Spreadsheet listing all differentially expressed genes in males exposed to the ALAN treatment. Gene = gene ID; base Mean = the mean of the normalized counts for all samples;log2FoldChange = Log 2 fold change in expression low-light relative to control; lfcMLE = unshrunken maximum likelihood estimates of Log2 fold change in expression; lfcSE = Standard Error of log2FoldChange; Stat = Wald statisic; pvalue; padj = *p* value adjusted after Benjamini-Hochberg; Annotation = functional annotation of the gene; Domain(s) = Interpro domain annotations. Genes that are differentially expressed with an absolute fold change greater than 2 (FC > 2) are shown in bold. (XLS 65 kb) Additional file 2: Table S2. Spreadsheet listing all differentially expressed genes in females exposed to the ALAN treatment based on adjusted *p*-values. Gene = gene ID; base Mean = the mean of the normalized counts for all samples; log2FoldChange = Log 2 fold change in expression low-light relative to control; lfcMLE = unshrunken maximum likelihood estimates of Log2 fold change in expression; lfcSE = Standard Error of log2FoldChange; Stat = Wald statisic; pvalue; padj = *p* value adjusted after Benjamini-Hochberg; Annotation = functional annotation of the gene; Domain(s) = Interpro domain annotations. (XLS 19 kb) Additional file 3: Table S3. Spreadsheet listing all overrepresented gene ontology terms associated with genes exhibiting decreased expression in males exposed to the ALAN treatment. GOBIP, gene ontology biological process identifier. (XLS 18 kb) Additional file 4: Table S4. Spreadsheet listing all differentially expressed gene s between male and female mosquitoes. GeneID = gene ID; base Mean = the mean of the normalized counts for all samples;log2FoldChange = Log 2 fold change in expression low-light relative to control; lfcMLE = unshrunken maximum likelihood estimates of Log2 fold change in expression; lfcSE = Standard Error of log2FoldChange; Stat = Wald statisic; pvalue; padj = *p* value adjusted after Benjamini-Hochberg; Annotation = functional annotation of the gene; Domain(s) = Interpro domain annotations. Genes with an absolute fold change of two are shown in bold. (XLS 2665 kb) Additional file 5: Table S5 Spreadsheet listing all overrepresented gene ontology terms associated with male-biased genes. GOBIP, gene ontology biological process identifier. (XLS 44 kb)

## Abbreviations

ALAN: artificial light at night
GO: Gene ontology
L:D: light:dark cycle
LED: light-emitting diode
ncRNA: non-coding RNA
PC: principal components
PCA: principal component analysis
UV: ultra violet
